# A comparison of oral health-related quality of life and satisfaction among patients undergoing root canal treatment or tooth extraction – A prospective controlled cohort study

**DOI:** 10.2340/aos.v83.42011

**Published:** 2024-10-14

**Authors:** Emma Wigsten, Emine Camci, Anna Levinsson, Thomas Kvist, Dan Sebring, EndoReCo, Thomas Davidson

**Affiliations:** a Department of Endodontology, Institute of Odontology, The Sahlgrenska Academy, University of Gothenburg, Gothenburg, Sweden; b Public Dental Service, Region Västra Götaland, Gothenburg, Sweden; c School of Public Health and Community Medicine, The Sahlgrenska Academy, University of Gothenburg, Gothenburg, Sweden; d Department of Epidemiology, Biostatistics and Occupational Health, Faculty of Medicine, McGill University, Montreal, Canada; e The Researchers within the Endodontic Research Collaboration in Scandinavia Contributed to This Study; f Department of Health, Medicine and Caring Sciences, Linköping University, Linköping, Sweden

**Keywords:** Endodontics, general dental practice, patient-centered care, questionnaire, root canal therapy

## Abstract

**Objective:**

This study aimed to evaluate the impact of root canal treatment on Oral Health-Related Quality of Life (OHRQoL) in general dental practice and compare it with tooth extraction. Additionally, patient satisfaction following tooth-preserving treatment was assessed.

**Material and methods:**

In all, 65 patients were recruited from 6 general dental clinics in Västra Götaland over 8 weeks, with 37 starting root canal treatment and 28 having extractions. Questionnaires, including Oral Health Impact Profile-14 (OHIP-14) and 9 questions assessing patient satisfaction, were administered at treatment initiation, and at 1, 6, and 12 months. The responses from both modalities were analysed using descriptive and analytical statistical methods.

**Results:**

The response rate ranged from 73.8% to 92.3%. Regarding OHRQoL, differences between the groups were few compared to baseline. However, significant improvements were observed in the extraction group at the 6- and 12-month follow-ups, in the ‘total score’, and the dimensions ‘pain’, ‘discomfort’, and ‘handicap’. Patient satisfaction was generally high, with cost being the least satisfactory item. Pain intensity remained consistently low.

**Conclusions:**

In this prospective cohort study few differences were found between the two treatment modalities. However, significant improvements were observed in the extraction group in several dimensions. The patient satisfaction regarding root canal treatment was considered high.

## Introduction

By default, the outcome of root canal treatment (RCT) has been assessed through clinical and radiographic evaluation of the biological condition of the treated tooth’s periradicular tissues. However, in recent decades, there has been an increased emphasis on patient-centred outcome measures, such as tooth survival [1]. Additionally, there has been increased interest in understanding how the treatment affects patients’ daily lives, often measured with the concept of quality of life (QoL) [2, 3], as well as how the patients experience the treatment itself in terms of satisfaction [4, 5].

The World Health Organization (WHO) defines the concept of QoL as *an individual’s perception of their position in life in the context of the culture and value systems in which they live and in relation to their goals, expectations, standards and concerns* [6]. In dentistry, the consequences of the various treatment modalities used to relieve or cure various oral diseases has been quantified by measuring the impact on QoL and more specifically Oral Health-Related Quality of Life (OHRQoL) [2, 3, 7, 8].

However, there are currently few prospective follow-up studies evaluating OHRQoL of RCT [9–11]. Ideally, the patients’ perceptions of their QoL is measured when the RCT is initiated and followed by the same instruments over time, when treatment is completed (root filled), when the tooth is in function, and at follow-ups [9–13]. It is fairly well-established that the preservation of the natural dentition with RCT has a positive impact on the OHRQoL [3, 9, 10, 12–15]. Therefore, if possible, RCT of a seriously damaged tooth seems preferable to extraction [14, 16].

Patient satisfaction is highly subjective and is based on the individual’s needs, expectations, and experiences [4, 5]. Dugas et al. [17] presented a questionnaire that addresses factors of perceived importance to patients receiving RCT, including such as pain during treatment and costs. Previous investigations suggest that patients are generally satisfied with their RCT [10, 16–19]. The most common sources of dissatisfaction concern cost and that the treatment can be time-consuming [16, 17, 19, 20].

Studies of patient-related outcomes of RCT conducted in the general dental practice are particularly few. In a prospective study of RCT, conducted at 20 different general dental care clinics in Sweden, approximately 50% of the patients who started RCT reported persistent mild pain still after 1 year [21]. Yet, the patients reported high satisfaction and indicated a willingness to undergo RCT again if suggested.

In a prior publication [20], we presented baseline data from a consecutively recruited cohort of patients who either started RCT or underwent tooth extraction. Sixty-five patients, treated by general dental practitioners at six public dental clinics, were included. After 1 month, an improvement in health-related quality of life (HRQoL) was observed among the patients who initiated RCT. However, no changes were observed in OHRQoL. Instead, more patients in the extraction group reported embarrassment at follow-up.

The aim of this study is to evaluate the OHRQoL and patient satisfaction of RCT, and compare it with tooth extraction in a patient cohort from general dental practice during a follow-up period of 1 year. Moreover, to test the null hypothesis that there are no significant differences between the two treatment modalities.

## Materials and methods

### Setting

This study is a prospective controlled cohort study. The population was recruited from six general dental care clinics affiliated with the Public Dental Service in the Region Västra Götaland, Sweden. These clinics provided geographic and socio-economic diversity, located in smaller cities and rural areas. The clinics also varied in size, with each clinic having between 3 and 10 general dental practitioners. A total of 47 practitioners participated in the recruitment process, which started in August and ended in December 2017. All clinics were connected to Swedish Social Insurance Agency (SSIA).

### Study population

The population and recruitment procedure have been described in detail previously [20]. The inclusion criteria were either starting RCT or undergoing tooth extraction within a predefined 8-week period. Additional inclusion criteria were being of legal age (≥ 18 years), ability to read and understand text in the Swedish language, and providing voluntary informed consent. The number of patients who declined participating or were ineligible because of language difficulties, or physical or mental illnesses was noted (*n* = 86).

### The Oral Health Impact Profile questionnaire

One of the most widely used questionnaires to measure OHRQoL is the Oral Health Impact Profile (OHIP). The questionnaire asks for the individual’s self-reported perception of any dysfunction, discomfort, and disability that can be attributed to an oral condition [2, 8]. The OHIP-14 is an abbreviated form [8] that contains 14 statements covering the 7 conceptual domains. The domains evaluate physical pain and disability, functional limitation, psychological discomfort and disability, social disability, and handicap.

A validated Swedish version of OHIP-14 was used (OHIP-14S) [22, 23]. Patients were asked to answer how frequently they experienced each of the 14 statements during the past month. The response options were based on a 5-point scale and were coded as follows: ‘very often’ (code 4), ‘fairly often’ (code 3), ‘occasionally’ (code 2), ‘hardly ever’ (code 1), and ‘never’ (code 0). The registered scores (codes) for each individual were summed to present the total score; the higher the total score (from 0 to a maximum of 56 points), the greater the impact on oral health and thus a poorer perceived OHRQoL [2, 8].

The questionnaire was distributed on four occasions. The first, representing baseline, was responded to at the appointment when treatment was started at the clinic [20]. Subsequently, the questionnaire was sent to the patients after 1, 6, and 12 months, respectively. If no response was received, a reminder was sent out after 3 weeks and with an additional telephone call if the questionnaire was still not returned. The mailouts took place between August 2017 and February 2018. All material was in Swedish.

Patient satisfaction and pain intensity was evaluated with 9 disease-specific questions constructed on the work by Dugas et al. [17]. The questions were evaluated on the Visual Analog Scale (VAS) (10 cm), graded from a positive (score 0) to a negative experience (score 10). The scales were constructed with different predefined end points. The patients’ experience of the treatment was evaluated in regards to perception of the aesthetics and function of the tooth, the overall experience, and finally the present pain intensity. The questions concerned only the patients who started the RCT.

### Statistical methods

Data were analysed as intention-to-treat using IBM^©^ SPSS^©^ Statistics Version 27 (SPSS Inc, Chicago, IL, USA). The recorded data are presented as mean, standard deviation (SD), median, and quantiles.

For the OHIP-14, the neutral ‘don’t know’/‘not applicable’ response option was treated as missing in the statistical analyses [8, 23], but for inclusion, patients were required to have given complete responses to the 14 statements. The responses were presented in its 7 dimensions. Patient satisfaction was measured with a ruler to the nearest decimal. Missing responses were excluded in the statistical analysis. When comparing over time, complete responses were required.

Following visual inspection of the OHIP-14 and VAS scores, it was determined that the assumption of a normal distribution of scores could not be made, and non-parametric tests were used. The Mann-Whitney *U*-test was used for comparison between groups of patients undergoing RCT and tooth extraction, respectively. For comparison within groups, the Wilcoxon Signed Rank test was used, and the difference between two and more occasions was tested using the Friedman’s Two-Way Analysis of Variance (ANOVA). All tests of significance were two-sided, and conducted at the 5% significance level (*P* < 0.05). Because of the study design, a sample size calculation was not performed.

### Ethical considerations

The Regional Ethical Committee in Gothenburg, Sweden, approved the study protocol in 2016 (dnr: 817–16). The study was outlined according to the STROBE and PROBE checklist and statements [24, 25]. All participating patients have given verbal and written informed consent.

## Results

In all, 65 patients participated in this prospective cohort study: 34 men (52.3%) and 31 women (47.7%), with a mean age of 55.5 years (SD = 15.1; range = 23–89 years). All third molars were excluded (*n* = 20). Out of the 65 patients, 37 started RCT and 28 underwent tooth extraction. Molar teeth predominated in both groups (*n* = 42, 64.6%). Preoperative patient- and tooth-specific factors as well as the cost-effectiveness between the two treatment modalities have been described in prior publications [20, 26]. The only statistically significant difference at baseline between the two groups was the number of previously root filled teeth (*P* = 0.03).

At the 1 year follow-up, 27 teeth in which RCT had been started, were root filled (73.0%) [26]. The remaining cases were either still under treatment (*n* = 2, 5.4%) or extracted (*n* = 8, 21.6%).

### The response rate and missing data

The total response rate of the questionnaire at baseline was 92.3% (*n* = 60), followed by 75.4% (*n* = 49), 73.8% (*n* = 48), and 81.5% (*n* = 53) at 1, 6, and 12 months. In all, 40 individuals (61.5%) responded to all four questionnaires, with 23 (57.5%) in the RCT group and 17 (42.5%) in the extraction group. Response rate regarding satisfaction with RCT at baseline was 81.1% (*n* = 30), followed by 59.5% (*n* = 22), 56.8% (*n* = 21), and 62.2% (*n* = 23) at 1, 6, and 12 months. The reasons for non-response are unknown.

### Oral health-related quality of life

The distribution of responses at baseline and the respective follow-up, along with the comparison between the two treatment modalities, is presented in Table 1. Overall, there were few statistically significant differences between the two groups in terms of perception of the impact on oral health, both regarding the total score and between the different dimensions.

**Table 1:** Distribution of responses to the Oral Health Impact Profile-14-S, comparing patients starting root canal treatment with a cohort of patients undergoing tooth extraction at baseline, that is, when treatment started, then after 1, 6, and 12 months. Presented with median, first and third quantile (Q1-Q3), and number of responses (n). Comparisons are made between treatment modalities at each given time period.

Time period:		Baseline			1-month follow-up			6-months follow-up			12-months follow-up		
Variable		RCT	Extraction	*P* ^3^	RCT	Extraction	*P* ^3^	RCT	Extraction	*P* ^3^	RCT	Extraction	*P* ^3^
Total score:^1^		8.96 (9.13) *n* = 24	9.47 (9.74) *n* = 19	0.668	5.82 (8.00) *n* = 22	12.00 (13.94) *n* = 15	0.092	6.00 (9.84) *n* = 23	5.15 (7.29) *n* = 20	0.592	6.29 (10.29) *n* = 28	4.45 (5.39) *n* = 20	0.444
Dimensions:^2^													
1	Functional limitation	0.0 (0.0–0.0) *n* = 30	0.0 (0.0–1.0) *n* = 22	0.547	0.0 (0.0–0.0) *n* = 24	0.0 (0.0–1.0) *n* = 18	**0.044**	0.0 (0.0–0.0) *n* = 24	0.0 (0.0–0.0) *n* = 21	0.839	0.0 (0.0–0.0) *n* = 30	0.0 (0.0–0.0) *n* = 21	0.586
2	Physical pain	2.0 (1.0–4.0) *n* = 30	3.0 (1.0–4.0) *n* = 26	0.528	2.0 (0.0–4.0) *n* = 29	2.0 (1.0–3.0) *n* = 19	0.532	0.0 (0.0–3.0) *n* = 27	1.0 (0.0–2.0) *n* = 21	0.478	1.00 (0.0–2.0) *n* = 29	0.5 (0.0–2.0) *n* = 22	0.951
3	Psychological discomfort	0.0 (0.0–2.0) *n* = 29	0.0 (0.0–2.0) *n* = 26	0.947	0.0 (0.0–2.0) *n* = 29	1.0 (0.0–4.0) *n* = 20	0.145	0.0 (0.0–1.0) *n* = 26	0.0 (0.0–1.0) *n* = 21	0.632	0.0 (0.0–1.0) *n* = 29	0.0 (0.0–0.0) *n* = 22	0.784
4	Physical disability	0.0 (0.0–1.0) *n* = 29	0.0 (0.0–1.0) *n* = 26	0.291	0.0 (0.0–1.0) *n* = 29	0.0 (0.0–1.0) *n* = 17	0.843	0.0 (0.0–1.0) *n* = 27	0.0 (0.0–1.0) *n* = 21	0.862	0.0 (0.0–1.0) *n* = 31	0.0 (0.0–1.0) *n* = 21	0.684
5	Psychological disability	1.0 (0.0–2.0) *n* = 29	0.0 (0.0–1.0) *n* = 25	0.293	1.0 (0.0–2.0) *n* = 27	1.0 (0.0–3.0) *n* = 19	0.366	0.0 (0.0–2.0) *n* = 26	0.0 (0.0–1.0) *n* = 21	0.844	0.0 (0.0–1.0) *n* = 30	0.0 (0.0–2.0) *n* = 22	0.295
6	Social disability	1.0 (0.0–2.0) *n* = 29	0.0 (0.0–2.0) *n* = 25	0.175	0.0 (0.0–1.0) *n* = 29	0.0 (0.0–3.0) *n* = 19	0.916	0.0 (0.0–1.0) *n* = 25	0.0 (0.0–0.0) *n* = 20	0.163	0.0 (0.0–2.0) *n* = 31	0.0 (0.0–1.0) *n* = 22	0.137
7	Handicap	1.0 (0.0–2.0) *n* = 29	1.0 (0.0–2.0) *n* = 24	0.887	0.0 (0.0–1.0) *n* = 28	0.0 (0.0–2.0) *n* = 19	0.502	0.0 (0.0–1.0) *n* = 25	0.0 (0.0–0.0) *n* = 20	0.370	0.0 (0.0–1.0) *n* = 30	0.0 (0.0–0.0) *n* = 22	**0.015**

*n*: number; Q1: first quantile; Q3: third quantile; RCT: root canal treatment; SD: standard deviation.

1 Total summary score, presented with mean and (SD): calculated for the patients (*n*) who responded all dimensions.

2 The dimensions [1–7] are presented with median and quantiles (Q1–Q3): calculated for the patients (*n*) who responded both questions for each dimension.

3 The *P*-values obtained by using the Mann-Whitney *U* test comparing the two treatment groups at each time period.

At the 1-year follow-up, the individuals who underwent RCT registered a greater handicap (*P* = 0.015). Most dimensions presented low scores (i.e*.* reported ‘never’; Table 1, Supplemental Table). The highest score was represented by ‘physical pain’, which applied to both treatment groups and all four occasions.

The difference between baseline and respective follow-up, both between and within the two treatment modalities, is presented in Table 2. Overall, there were no statistically significant differences between the two groups from baseline to the respective follow-up. Most differences within treatment groups were small and not statistically significant. However, at 6-months follow-up for the individuals who underwent tooth extraction, an improved total median score was registered (*P* = 0.015), as well as a reduction in physical pain (*P* = 0.015) and psychological discomfort (*P* = 0.036). At 1-year follow-up, an improvement was registered for both groups regarding physical pain (*P* = 0.010 and *P* = 0.009), and the extraction group experienced reduced handicap (*P* = 0.009) compared to baseline.

**Table 2:** Comparison of responses to the Oral Health Impact Profile-14-S, between patients starting root canal treatment with a cohort of patients undergoing tooth extraction at baseline, that is, when treatment started, then after 1, 6, and 12 months. Comparisons are made between baseline and respective follow-up, between^3^ and within each treatment group^4^

Time period:		1-month follow-up			6-months follow-up			12-months follow-up		
Variable		*P* ^3^	(*n*)	*P* ^4^	*P^3^*	(*n*)	*P* ^4^	*P* ^3^	(*n*)	*P* ^4^
Total score:^1^		0.494	RCT (*n* = 14) Extraction (*n* = 12)	0.953 0.637	0.270	RCT (*n* = 15) Extraction (*n* = 14)	0.575 **0.015**	0.444	RCT (*n* = 17) Extraction (*n* = 14)	0.422 0.100
Dimensions:^2^										
1	Functional limitation	0.286	RCT (*n* = 19) Extraction (*n* = 16)	0.480 0.102	0.460	RCT (*n* = 20) Extraction (*n* = 18)	0.916 0.168	0.313	RCT (*n* = 24) Extraction (*n* = 18)	1.000 0.395
2	Physical pain	0.408	RCT (*n* = 23) Extraction (*n* = 18)	0.485 0.676	0.410	RCT (*n* = 22) Extraction (*n* = 19)	0.064 **0.015**	0.430	RCT (*n* = 24) Extraction (*n* = 20)	**0.010** **0.009**
3	Psychological discomfort	0.497	RCT (*n* = 23) Extraction (*n* = 18)	0.179 0.757	0.405	RCT (*n* = 21) Extraction (*n* = 19)	0.639 **0.036**	0.333	RCT (*n* = 23) Extraction (*n* = 20)	0.758 0.064
4	Physical disability	0.921	RCT (*n* = 23) Extraction (*n* = 16)	0.430 0.438	0.936	RCT (*n* = 21) Extraction (*n* = 19)	0.609 0.506	0.360	RCT (*n* = 23) Extraction (*n* = 19)	0.932 0.396
5	Psychological disability	0.450	RCT (*n* = 21) Extraction (*n* = 17)	0.458 0.194	0.534	RCT (*n* = 20) Extraction (*n* = 18)	0.441 0.762	0.319	RCT (*n* = 22) Extraction (*n* = 19)	0.433 0.401
6	Social disability	0.492	RCT (*n* = 22) Extraction (*n* = 18)	0.378 0.755	0.594	RCT (*n* = 19) Extraction (*n* = 17)	0.075 0.084	0.831	RCT (*n* = 23) Extraction (*n* = 19)	0.480 0.222
7	Handicap	0.672	RCT (*n* = 22) Extraction (*n* = 16)	0.278 0.958	0.844	RCT (*n* = 21) Extraction (*n* = 16)	0.054 0.058	0.181	RCT (*n* = 23) Extraction (*n* = 18)	0.257 **0.009**

n: number; RCT: root canal treatment.

1 The *P*-value of total summary score was calculated for the patients (*n*) who responded all dimensions and both time periods: baseline and respectively follow-up.

2 The dimensions [1–7] are calculated for the individuals (*n*) who responded both questions for each dimension and both time periods: baseline and respectively follow-up.

3 *P*-values obtained using the Mann-Whitney *U* test analysing the difference between baseline and respective follow-up comparing the two treatment groups.

4 *P*-values obtained using the Wilcoxon Signed Rank Test analysing the difference between baseline and respective follow-up within each treatment group. Presented with the number (*n*) of included responses.

### Patient satisfaction with root canal treatment

The distribution of responses and the comparisons between baseline and respective follow-up are presented in Table 3. Overall, most differences were small and not statistically significant. At 1-year follow-up, the patients who started RCT registered an increased perception of intraoperative pain (*P* = 0.013 [difference between baseline and follow-up] and *P* = 0.048 [the difference over time].

**Table 3:** Patient satisfaction with root canal treatment during four different time periods. At baseline, that is, when treatment started, then after 1, 6, and 12 months. Presented with median, first and third quantiles (Q1–Q3), and number of responses (n). Comparisons are made between baseline and respective follow-up.

Time period		Baseline^1^	1-month follow-up^1^			6-months follow-up^1^			12-months follow-up^1^			Total
Variable				*P* ^2^	*P^3^*		*P* ^2^	*P* ^3^		*P* ^2^	*P* ^3^	*P* ^4^
1	Pleasantness	1.9 (0.4–6.2) *n* = 26	4.7 (0.5–7.2) *n* = 16	0.777	0.543	4.6 (0.7–6.3) *n* = 21	0.102	0.543	5.1 (0.5–7.9) *n* = 15	0.170	0.224	0.647
2	Intraoperative pain	0.7 (0.4–4.7) *n* = 30	1.2 (0.4–5.7) *n* = 19	0.129	0.213	1.1 (0.5–4.7) *n* = 21	0.209	0.143	1.6 (0.4–5.0) *n* = 18	**0.013**	**0.048**	0.184
3	Time involved	1.8 (0.5–5.7) *n* = 29	3.1 (1.0–7.1) *n* = 18	0.089	0.124	3.2 (0.8–7.0) *n* = 21	0.103	0.242	2.7 (1.1–6.0) *n* = 17	0.114	0.272	0.421
4	Cost	4.8 (2.2–9.3) *n* = 21	7.5 (0.8–9.1) *n* = 11	0.593	0.535	5.2 (0.6–8.1) *n* = 19	1.000	0.301	6.0 (1.3–7.2) *n* = 9	0.635	0.679	0.774
5	Worth the cost	1.0 (0.3–3.2) *n* = 22	1.4 (0.3–5.4) *n* = 12	0.432	0.846	0.7 (0.3–5.7) *n* = 20	0.789	0.699	0.9 (0.5–2.8) *n* = 11	0.906	0.846	0.984
6	Postoperative aesthetics	1.5 (0.6–4.2) *n* = 25	1.0 (0.3–2.6) *n* = 16	0.134	0.248	1.1 (0.5–4.1) *n* = 20	0.328	0.099	3.1 (0.7–5.6) *n* = 14	0.345	0.869	0.168
7	Chewing ability	1.0 (0.3–1.9) *n* = 24	0.7 (0.4–2.0) *n* = 15	0.569	0.160	0.8 (0.5–1.8) *n* = 21	0.381	0.509	0.9 (0.5–2.8) *n* = 13	0.480	0.804	0.191
8	General satisfaction	0.4 (0.3–1.8) *n* = 26	0.5 (0.4–1.9) *n* = 16	0.315	0.635	0.9 (0.4–3.8) *n* = 21	0.080	0.752	1.1 (0.4–4.8) *n* = 17	0.348	0.268	0.695
9	Pain intensity (VAS)	0.6 (0.4–1.4) *n* = 27	0.7 (0.3–2.1) *n* = 17	0.924	1.000	0.6 (0.3–2.5) *n* = 21	0.480	0.820	0.8 (0.4–2.2) *n* = 15	0.221	0.704	0.974

*n*: number; Q1: first quantile; Q3: third quantile; VAS: visual analog scale.

1 Number of patients (*n*) who responded the question at baseline and respective follow-up. The patients who had their tooth extracted were instructed not to respond the questionnaire. The statistical analysis required responses at baseline and the respective follow-up.

2 *P*-values obtained using the Wilcoxon Signed Rank test, analysing the difference between baseline and respective follow-up.

3 *P*-values obtained using the Friedman’s Two-Way ANOVA, analysing the difference from baseline to respective follow-up.

4 *P*-values obtained using the Friedman’s Two-Way ANOVA, comparing the difference from baseline through all follow-ups.

The majority of the median values were less than 2.0 (*n* = 25, 69.4%; Table 3). The questions that presented the highest satisfaction with RCT were: ‘general satisfaction’, ‘chewing ability’, and the experience that the treatment was ‘worth the cost’ across all four occasions. The three factors that registered the greatest dissatisfaction were: ‘cost’, ‘pleasantness’, and time spent in the chair during the four occasions. Cost presented the highest value on the first three occasions, while ‘pleasantness’ presented the highest value at the 1-year follow-up. The lowest value was observed for ‘general satisfaction’ on the first two occasions, followed by ‘chewing ability on the last two occasions, as well as whether it was ‘worth the cost’.

### Estimating pain intensity

At baseline, 25 patients (92.6%) reported pain (VAS > 0) with median pain intensity of 0.6 (0.4–1.4; Table 3). Pain intensity was categorised as follows: no pain (VAS = 0), mild pain (VAS > 0–3), and moderate to severe pain (VAS > 3). At baseline, 2 patients (7.4%) reported no pain, 22 patients (81.5%) reported mild pain, and 3 patients (11.1%) reported moderate to severe pain. After 1 month, 2 patients (9.1%) reported no pain, 18 (81.8%) reported mild pain, and 2 (9.1%) reported mild to severe pain. At 6 months, 2 patients (9.5%) reported no pain, 16 (76.2%) reported mild pain, and 3 (14.3%) reported moderate to severe pain. Finally, at 1-year follow-up, 2 patients (9.1%) reported no pain, 17 patients (77.3%) reported mild pain, and 3 patients (13.6%) reported moderate to severe pain. From baseline to the 1-year follow-up, the present pain intensity continued to be low (VAS < 3) with no statistically significant differences over the time periods (Table 3).

## Discussion

This prospective study assessed the patient-centred outcome of RCT or tooth extraction using OHIP-14-S and a patient satisfaction questionnaire. Satisfaction with RCT was generally high. Pain intensity remained consistently mild throughout the study with no significant changes over time. Patients undergoing extraction showed reduction of pain, discomfort, and handicap at 1- and 12-months. In summary, there were limited differences in OHRQoL between patients that received RCT and those that had tooth extraction at baseline over the 1-year follow-up period. However, the null hypothesis was rejected as significant differences were observed.

Previous follow-up studies have reported a positive impact on OHRQoL as assessed using OHIP-14 [9, 10, 12, 13] and the endodontic-specific instrument OHIP-17 [19, 27]. However, our previous study on 1-month follow-up [20] and the present 1-year follow-up did not reveal such a positive change among patients undergoing RCT. This divergence may be attributed to variations in inclusion criteria and service providers. The patients in this study, in general, did not report high levels of pain at baseline which in other settings may be more common. The fact that our patients were recruited and treated by general dental practitioners may also have influenced the results. Moreover, the lack of a standardised method for analysing outcomes may complicate comparisons between studies, even when employing similar methodologies.

On the other hand, there may in fact be no major difference in QoL after an RCT, in particular if no or only mild pain was involved at start. Maybe, the instruments intended to measure OHRQoL may not be appropriately aligned with the essential patient-related outcomes of RCT. The original instrument was designed to assess the impact of oral health on older individuals with tooth loss [2], which may explain why a positive effect was more obvious among individuals undergoing extraction.

The high patient satisfaction aligns with previous studies conducted at university clinics and with other dental service providers [16, 17, 19], with the difference that most other studies started recruitment of patients when RCT has been completed with a root filling. There are few studies that have studied satisfaction from the initiation of treatment and over an extended period of time [20, 21]. In another population, also conducted in general dental practice, we observed that the majority of patients were satisfied, even though one-third of the treatments did not result in a root filling within 1 to 3 years, and fewer than two-thirds of molars underwent root filling [21]. It seems that most patients prefer to retain their teeth if possible [16, 21]. Despite the cost being a negative factor [16, 17, 19, 21], most still strive to preserve their dentition and consider the treatment to be worth the cost [21].

During the four occasions, from baseline and throughout the follow-ups, most individuals reported mild pain, which persisted. The prevalence of pain differs from previous studies where most individuals are symptom-free after their RCT. In a study conducted by Jonsson Sjögren et al. [28] at 23 general dental clinics in Örebro, Sweden, approximately 5% of the patients reported pain or discomfort from their root filled teeth, which is consistent with the findings from the systematic review and meta-analysis carried out by Nixdorf et al. [29].

In contrast, in an original cohort of 243 patients, 50% (n = 59) of the respondents reported pain, mostly of mild intensity (76.3%), one year after initiating RCT [21]. The variation in outcomes may be attributed to differences in inclusion criteria, the timing of assessment after RCT, or possibly differences among patients or/and service providers. Given this discrepancy with previously published data continued follow-up of the cohort is warranted.

This study displays several noteworthy strengths. Firstly, it presents two distinct cohorts, primarily differentiated by the number of root filled teeth at baseline [20], and follows them over time. Additionally, the recruitment of participants from six general dental practices, where the majority of dental treatments are performed [30], ensures that the findings are based on real-world scenarios, with diagnoses and treatments unaffected by participation in the study. Furthermore, the relatively high response rate to the overall questionnaire enhances the reliability of the collected data.

The study’s 1-year follow-up, conducted on four different occasions, theoretically offers a comprehensive longitudinal perspective of how dental treatments impact patients’ lives. However, the comparison between the groups may not be fair because the patient and dentist had voluntarily chosen which treatment, RCT or extraction, would be preferred in the individual case. For a fair comparison, RCT or extraction should be selected by random. That study design, to our knowledge, hasn’t been adopted for teeth in need of RCT event, other than when it comes to endodontic revision treatment versus extraction and implants [31]. A randomised study on RCT or extraction and replacement evaluated by patient-related outcomes as well as a cost-effective analysis is desirable and potentially useful, but at the same time, there are substantial practical and ethical complicating circumstances to consider.

Despite its strengths and limitations, the study revealed few differences between the treatment modalities and within the groups over time. Emphasising patient-centred outcomes is important and should be included in clinical research assessing the impact of treatment on individuals’ daily lives and treatment experience. Notably, high levels of patient satisfaction, despite associated costs, suggest that tooth-preserving treatments such as RCT are considered worthwhile by patients. This reinforces the significance of involving patients in treatment decisions and prioritising tooth retention whenever feasible.

However, the lack of prospective clinical follow-up studies in general dental practice underscores the need for continued research in this area. Addressing existing knowledge gaps through further follow-up studies is essential for enhancing our understanding of both patient experiences and treatment outcomes in real-world dental settings. Future research should aim to incorporate patient-reported outcomes and consider the long-term implications of different treatment approaches to inform clinical practice effectively.

## Conclusion

In this prospective cohort study, the differences in OHRQoL over 1 year between patients initiating RCT and those undergoing tooth extraction were few. However, further improvement was observed in the group treated with extraction. The patient satisfaction regarding RCT was considered as high.

## Supplementary Material

A comparison of oral health-related quality of life and satisfaction among patients undergoing root canal treatment or tooth extraction – A prospective controlled cohort study

## Data Availability

The data that support the findings of this study are available from the corresponding author, EW, upon reasonable request.

## Geolocation information

The study was conducted in Region Västra Götaland, Sweden.
