# Supplementary material for: A comparison of oral health-related quality of life and satisfaction among patients undergoing root canal treatment or tooth extraction – A prospective controlled cohort study
Source: Acta Odontol Scand. 2024 Oct 14;83:42011. doi: 10.2340/aos.v83.42011 (PMC11487112; doi:10.2340/aos.v83.42011)
Supplement: A comparison of oral health-related quality of life and satisfaction among patients undergoing root canal treatment or tooth extraction – A prospective controlled cohort study [file AOS-83-42011-s1.pdf]

Supplementary material has been published as submitted. It has not been copyedited or typeset by Acta Odontologica Scandinavica.

Supplemental table - Distribution of respondents' responses to OHIP-14, and in comparison, between root canal treatment (*n* = 37) and extraction (*n* = 28) at baseline and follow-ups.

		RCT	Extraction	RCT	Extraction	RCT	Extraction
		Never (score 0)		Hardly ever - very often (score 1-4)		Fairly often - very often (score 3-4)	
Functional limitation, n (%):							
Q1	Baseline	27 (90.0)	18 (75.0)	3 (10.0)	6 (25.0)	0 (0.0)	1 (4.2)
	1 month	22 (88.0)	11 (61.1)	3 (12.0)	7 (38.9)	1 (4.0)	1 (5.6)
	6 months	22 (91.7)	18 (85.7)	2 (8.3)	3 (14.3)	1 (4.2)	1 (4.8)
	12 months	27 (90.0)	19 (86.4)	3 (10.0)	3 (13.6)	0 (0.0)	2 (9.1)
Q2	Baseline	26 (83.9)	21 (87.5)	5 (16.1)	3 (12.5)	1 (3.2)	0 (0.0)
	1 month	23 (88.5)	15 (83.3)	3 (11.5)	3 (16.7)	1 (3.8)	1 (5.6)
	6 months	22 (84.6)	17 (81.0)	4 (15.4)	4 (19.0)	2 (7.7)	1 (4.8)
	12 months	28 (90.3)	18 (85.7)	3 (9.7)	3 (14.3)	3 (9.7)	1 (4.8)
Physical pain, n (%):							
Q3	Baseline	6 (19.4)	5 (18.5)	25 (80.6)	22 (81.5)	6 (19.4)	5 (18.5)
	1 month	11 (37.9)	6 (31.6)	18 (62.1)	13 (68.4)	4 (13.8)	2 (10.5)
	6 months	15 (55.6)	14 (66.7)	12 (44.4)	7 (33.3)	2 (7.4)	0 (0.0)
	12 months	18 (58.1)	15 (68.2)	13 (41.9)	7 (31.8)	2 (6.5)	0 (0.0)
Q4	Baseline	16 (53.3)	9 (33.3)	14 (46.7)	18 (66.7)	5 (16.7)	3 (11.1)
	1 month	14 (48.3)	5 (25.0)	15 (51.7)	15 (75.0)	5 (17.2)	4 (20.0)
	6 months	17 (63.0)	9 (42.9)	10 (37.0)	12 (57.1)	2 (7.4)	1 (4.8)
	12 months	19 (65.5)	12 (54.5)	10 (34.5)	10 (45.5)	1 (3.4)	1 (4.5)
Psychological discomfort, n (%):							
Q5	Baseline	21 (72.4)	17 (63.0)	8 (27.6)	10 (37.0)	1 (3.4)	4 (14.8)
	1 month	22 (75.9)	8 (40.0)	7 (24.1)	12 (60.0)	1 (3.4)	1 (5.0)
	6 months	20 (76.9)	16 (76.2)	6 (23.1)	5 (23.8)	0 (0.0)	0 (0.0)
	12 months	23 (79.3)	18 (81.8)	6 (20.7)	4 (18.2)	1 (3.4)	0 (0.0)
Q6	Baseline	17 (56.7)	18 (69.2)	13 (43.3)	8 (30.8)	3 (10.0)	3 (11.5)
	1 month	17 (58.6)	12 (60.0)	12 (41.4)	8 (40.0)	1 (3.4)	2 (10.0)
	6 months	20 (74.1)	15 (71.4)	7 (25.9)	6 (28.6)	1 (3.7)	0 (0.0)
	12 months	23 (76.7)	17 (77.3)	7 (23.3)	5 (22.7)	1 (3.3)	0 (0.0)
Physical disability, n (%):							
Q7	Baseline	22 (75.9)	15 (57.7)	7 (24.1)	11 (42.3)	2 (6.9)	2 (7.7)
	1 month	19 (65.5)	13 (72.2)	10 (34.5)	5 (27.8)	1 (3.4)	3 (16.7)
	6 months	21 (77.8)	16 (76.2)	6 (22.2)	5 (23.8)	1 (3.7)	1 (4.8)
	12 months	23 (74.2)	15 (71.4)	8 (25.8)	6 (28.6)	2 (6.5)	2 (9.5)
Q8	Baseline	24 (77.4)	20 (76.9)	7 (22.6)	6 (23.1)	0 (0.0)	0 (0.0)
	1 month	26 (89.7)	14 (73.7)	3 (10.3)	5 (26.3)	1 (3.4)	1 (5.3)
	6 months	24 (88.9)	18 (85.7)	3 (11.1)	3 (14.3)	1 (3.7)	0 (0.0)
	12 months	28 (90.3)	19 (86.4)	3 (9.7)	3 (13.6)	1 (3.2)	1 (4.5)

Psychological disability, n (%):							
Q9	Baseline	14 (43.8)	15 (57.7)	18 (56.3)	11 (42.3)	4 (12.5)	1 (3.8)
	1 month	13 (44.8)	10 (50.0)	16 (55.2)	10 (50.0)	4 (13.8)	1 (5.0)
	6 months	17 (65.4)	15 (71.4)	9 (34.6)	6 (28.6)	2 (7.7)	0 (0.0)
	12 months	20 (64.5)	15 (68.2)	11 (35.5)	7 (31.8)	1 (3.2)	1 (4.5)
Q10	Baseline	23 (79.3)	19 (73.1)	6 (20.7)	7 (26.9)	0 (0.0)	2 (7.7)
	1 month	24 (88.9)	11 (57.9)	3 (11.1)	8 (42.1)	0 (0.0)	1 (5.3)
	6 months	21 (80.8)	16 (76.2)	5 (19.2)	5 (23.8)	1 (3.8)	1 (4.8)
	12 months	26 (86.7)	13 (59.1)	4 (13.3)	9 (40.9)	1 (3.3)	2 (9.1)
Social disability, n (%):							
Q11	Baseline	11 (36.7)	14 (56.0)	19 (63.3)	11 (44.0)	3 (10.0)	2 (8.0)
	1 month	16 (55.2)	12 (60.0)	13 (44.8)	8 (40.0)	2 (6.9)	1 (5.0)
	6 months	15 (60.0)	16 (80.0)	10 (40.0)	4 (20.0)	3 (12.0)	1 (5.0)
	12 months	17 (54.8)	16 (72.7)	14 (45.2)	6 (27.3)	1 (3.2)	0 (0.0)
Q12	Baseline	19 (61.3)	19 (73.1)	12 (38.7)	7 (26.9)	2 (6.5)	0 (0.0)
	1 month	24 (82.8)	13 (68.4)	5 (17.2)	6 (31.6)	2 (6.9)	1 (5.3)
	6 months	22 (84.6)	18 (90.0)	4 (15.4)	2 (10.0)	2 (7.7)	0 (0.0)
	12 months	24 (77.4)	21 (95.5)	7 (22.6)	1 (4.5)	2 (6.5)	0 (0.0)
Handicap, n (%):							
Q13	Baseline	14 (45.2)	10 (40.0)	17 (54.8)	15 (60.0)	4 (12.9)	2 (8.0)
	1 month	18 (62.1)	11 (57.9)	11 (37.9)	8 (42.1)	2 (6.9)	1 (5.3)
	6 months	17 (68.0)	16 (80.0)	8 (32.0)	4 (20.0)	2 (8.0)	0 (0.0)
	12 months	16 (51.6)	19 (86.4)	15 (48.4)	3 (13.6)	1 (3.2)	0 (0.0)
Q14	Baseline	23 (79.3)	20 (80.0)	6 (20.7)	5 (20.0)	1 (3.4)	1 (4.0)
	1 month	24 (85.7)	14 (73.7)	4 (14.3)	5 (26.3)	1 (3.6)	1 (5.3)
	6 months	21 (84.0)	18 (90.0)	4 (16.0)	2 (10.0)	0 (0.0)	0 (0.0)
	12 months	27 (90.0)	22 (100.0)	3 (10.0)	0 (0.0)	1 (3.3)	0 (0.0)

Q, question; RCT, root canal treatment.
